# Granzyme B Expression in Conjunctiva of Patients with Pterygium

**DOI:** 10.3390/ijms25168679

**Published:** 2024-08-09

**Authors:** Yoojin Choi, Isa Samad, Harshini Chakravarthy, Joanne Matsubara, David J. Granville, Sonia N. Yeung

**Affiliations:** 1 Faculty of Medicine, University of British Columbia, Vancouver, BC V6T 1Z4, Canada; 2 Department of Ophthalmology and Visual Sciences, University of British Columbia, Vancouver, BC V5Z 3N9, Canada; 3 ICORD Centre and Department of Pathology and Laboratory Medicine, University of British Columbia, Vancouver, BC V6T 2B5, Canada

**Keywords:** pterygium, conjunctiva, Granzyme B, tryptase, mast cells, ocular disease

## Abstract

Pterygium is often associated with chronic ultraviolet (UV) radiation exposure and characterized by the overgrowth of conjunctiva and extracellular matrix (ECM) remodeling. Notably, several studies in the skin have demonstrated that chronic UV radiation can upregulate Granzyme B (GrB) expression and increase ECM degradation. The aim of this study was to compare GrB expression between pterygium and healthy controls and to further link this GrB expression to mast cells. Post-mortem pterygium tissues and conjunctival tissues from age-matched controls were used to assess GrB expression via immunofluorescence and microscopy. We found a significantly higher density of GrB+ cells from pterygium specimens compared to healthy controls. Furthermore, many of the GrB+ cells in pterygium specimens co-expressed tryptase, a mast cell marker. These findings suggest a role for conjunctival mast cell-secreted GrB in the pathogenesis of pterygium and highlight GrB as a possible therapeutic target in delaying or halting pterygium progression.

## 1. Introduction

Pterygium is an ocular surface disease often linked to chronic ultraviolet (UV) radiation exposure [1]. Its prevalence varies across the world ranging from 1.3% in Tehran to 39% in China [2,3]. The pathogenesis of pterygium is characterized by a benign fibrovascular growth of the conjunctiva and aberrant extracellular matrix (ECM) remodeling. The growth can extend beyond the periphery of the cornea into the visual axis, leading to visual impairment or rarely, vision loss [4]. Currently, the gold-standard treatment for pterygium is surgical excision combined with a conjunctival autograft [5]. However, recurrent pterygium and an increased size of pterygium are associated with an increased risk of recurrence after surgical excision [6]. Other than primary prevention by reducing chronic UV exposure, there is no other means for risk reduction for the development and progression of pterygium. Therefore, alternative methods to reduce or prevent disease progression or recurrence are needed [7].

Granzyme B is a serine protease that is predominantly expressed by immune cells and accumulates in the extracellular milieu during prolonged inflammation. Previous studies have reported increased accumulation of GrB-secreting CD8^+^ T cells in pterygium [8,9,10]. However, GrB is also secreted by cells of the innate immune system, such as NK cells, macrophages, and mast cells [11,12,13]. Chronic UV exposure upregulates GrB expression and secretion from keratinocytes and dermal mast cells, resulting in fibronectin and decorin degradation and impaired matrix remodeling [14,15]. Similar to what is known about GrB in the skin, we recently reported that GrB also cleaves ECM proteins and impairs remodeling in age-related macular degeneration [16]. Furthermore, several groups have implicated UV exposure in the pathogenesis of pterygium via activation of inflammasomes [17], induction of proinflammatory cytokines [18], and upregulation of matrix metalloproteinase (MMP) expression [19]. Interestingly, GrB induces IL-8 secretion from epithelial cells [20], while GrB-generated fibronectin cleavage fragments induce MMP expression in dermal fibroblasts, leading to increased collagen degradation in aged skin [14]. Because pterygium is associated with UV exposure, we hypothesized that GrB is upregulated in pterygium. Further, we hypothesized that mast cells contribute towards GrB expression in pterygium, much alike what has been observed in the skin.

## 2. Results

### 2.1. Granzyme B Expression in Human Conjunctiva

**Figure 1:**
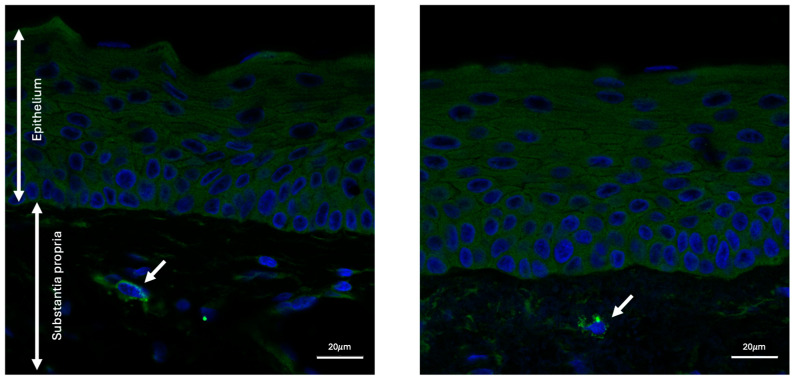
Representative images of conjunctiva from pterygium specimens stained for Granzyme B (green) and DAPI (blue) imaged at 40×. Granzyme B-positive cells are visible in the stromal layer as indicated by the arrows.

Immuno-stained sections from donors with and without pterygium were visualized to identify GrB. Both pterygium and age-matched control samples exhibited GrB positivity that was granular in appearance. In both pterygium and age-matched control specimens, most GrB-positive signals were localized to the stromal layer of the conjunctiva, under the epithelial layer (Figure 1).

### 2.2. Higher Density of Granzyme B-Positive Cells in Pterygium

**Figure 2:**
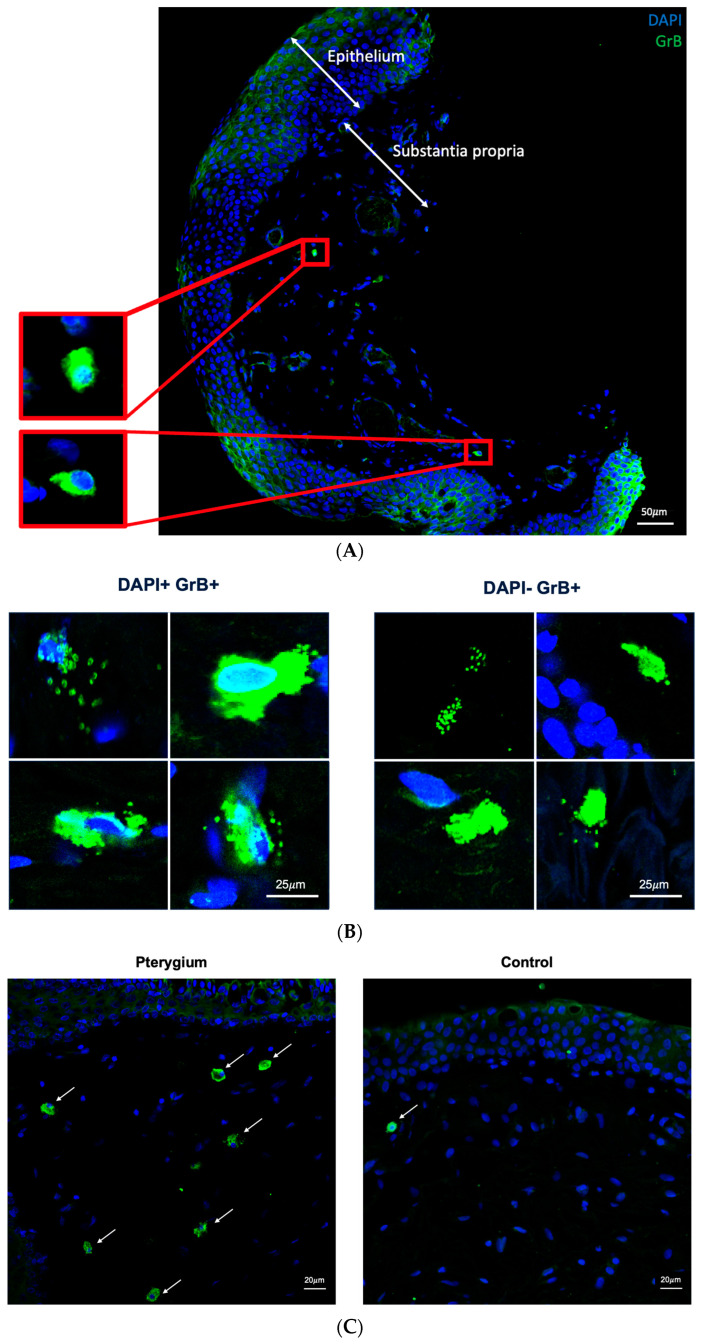

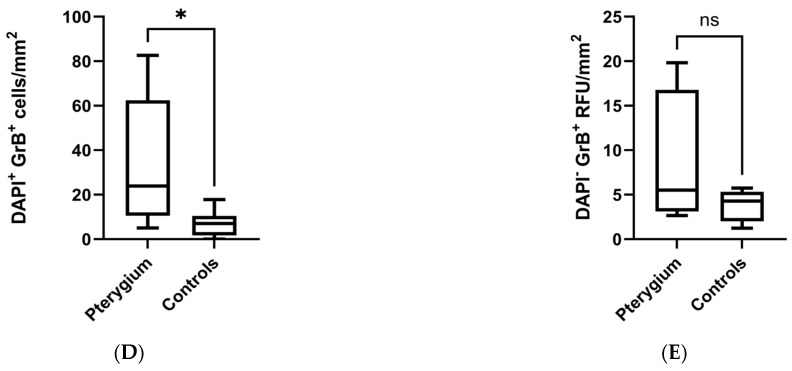
Higher density of Granzyme B-positive cells in pterygium than healthy controls using tiling at 40×. (**A**) Representative image of pterygium tissue (**B**) Representative images of DAPI-associated and DAPI-negative, Granzyme B-positivity (**C**) Representative images of Granzyme B-positive cells in pterygium and controls (**D**) DAPI+ GrB+ cells in pterygium and controls. (**E**) DAPI− GrB+ RFU in pterygium and controls. Mann–Whitney. * shows *p* < 0.05. RFU stands for relative fluorescence units; ns stands for not significant. Blue staining for DAPI and green staining for Granzyme B in all images. Arrows point to Granzyme B-positive cells.

After confirming the presence of GrB in both pterygium and control specimens, we then used a quantitative approach by tiling the tissues at 40× as described in the Section 4 (Figure 2A). Notably, there were two types of GrB-positive signals: DAPI-associated and DAPI-free (Figure 2B). DAPI-associated, GrB-positive signals likely represent intracellular GrB that has not been released. Conversely, DAPI-free, GrB-positive signals likely represent degranulation products, meaning GrB that has been released from the cell of origin. As for DAPI-associated, GrB-positive signals, there was a significantly higher density of signals from pterygium specimens compared to healthy controls (Figure 2C; 5.02–82.61 cells/mm^2^ vs. 0–17.76 cells/mm^2^; *p* = 0.021). Conversely, the density of DAPI-free GrB-positive signals were comparable between pterygium and control specimens (Figure 2D; 2.66–19.83 signals/mm^2^ vs. 1.23–5.74 signals/mm^2^; *p* = 0.248).

### 2.3. Granzyme B Expression and Age

**Figure 3:**
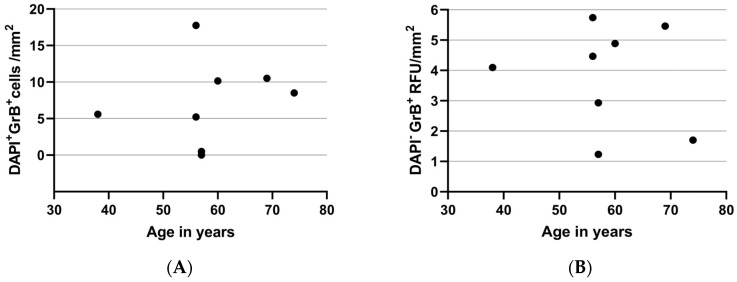
No correlation between age and (**A**) DAPI+ GrB+ cells or (**B**) DAPI− GrB+ RFU. Spearman’s correlation test. RFU stands for relative fluorescence units.

UV-induced, MMP-mediated degradation of type I collagen fibrils contributes to aging in the skin [21]. As GrB-mediated fibronectin fragments increase MMP1 production and GrB-mediated decorin cleavage permits MMP1 access to collagen and degradation [14], we assessed for any correlation between GrB and age in our healthy human conjunctival specimens. Interestingly, we did not see a correlation between the age of the donors and GrB expression (Figure 3).

### 2.4. Granzyme B and Tryptase Co-Expression in Pterygium

**Figure 4:**
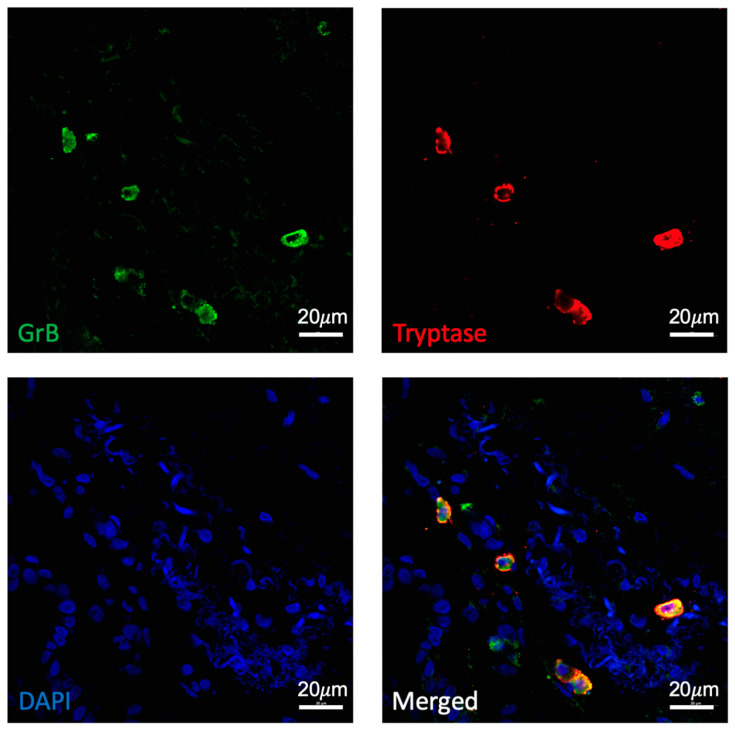
Co-expression of GrB (green) and tryptase (red) in pterygium imaged at 40×. DAPI in blue.

Given the finding that pterygium is associated with a significantly higher density of GrB expression, we were interested in identifying the type of immune cell responsible for GrB production in human conjunctiva. One study previously reported that angiogenesis in pterygium tissues was strongly correlated with tryptase-positive mast cell count [22]. Moreover, other studies demonstrated an increased number of mast cells in pterygium compared to normal conjunctiva [23,24,25]. As such, we used a well-validated marker of mast cells, tryptase, to assess whether mast cells are implicated in GrB production in pterygium. Indeed, we observed that many GrB-positive cells in pterygium specimens also expressed tryptase (Figure 4).

## 3. Discussion

Our study demonstrates that pterygium tissues have significantly higher density of GrB expression when compared to healthy conjunctival tissues. GrB-positive cells in pterygium were primarily located in the tissue stromal layers. Interestingly, many of the GrB-positive cells in pterygium specimens also expressed tryptase, a mast cell marker. These findings suggest a role that GrB from conjunctival mast cells plays in the pathogenesis of pterygium and highlights GrB as a possible therapeutic target in delaying or halting pterygium progression.

Proteases have consistently been implicated in the pathogenesis of pterygium [19,26,27,28,29]. However, inhibiting MMPs as a therapeutic target for ocular disease has largely been unsuccessful, in part due to the critical roles MMPs play in maintaining corneal homeostasis. Furthermore, as many of the 24 MMPs exert critical roles in the regulation of inflammation [30], global inhibition of MMPs would be contraindicated and could exacerbate inflammation. In searching for another therapeutic target, our team was interested in GrB, a serine protease that indirectly augments MMP expression through the fragmentation of fibronectin. Further, GrB is present at very low levels in young healthy adults [31], and reduced GrB is associated with healthy aging in the absence of any obvious adverse events in ApoE-KO mice [32]. These results suggest that inhibiting GrB is unlikely to cause adverse effects under normal physiological conditions and support the potential for GrB to be a safe therapeutic target in ocular diseases such as pterygium.

Although the potential role GrB plays in the pathogenesis of pterygium is largely unknown, parallels can be drawn from studies that assessed the pathogenesis of other UV-associated diseases, particularly in the skin. For instance, chronic UV-exposed murine skin has increased GrB expression compared to skin from non-irradiated mice [14]. Further, GrB deficiency in this mouse model was associated with protection against the loss of collagen density in UV-treated skin. Similarly, a study using human keratinocytes also demonstrated increased GrB expression after UV irradiation [15]. As such, GrB may be implicated in the pathogenesis of pterygium via UV-associated induction.

In our study, many of the GrB-positive cells in pterygium specimens also expressed tryptase, a marker of mast cells. This finding is in line with previous work. For example, one study found that there was a significant increase in the number of mast cells in the skin of mice after UV exposure as compared to control mice [14]. Similarly, other studies demonstrated increased mast cells in pterygium compared to healthy controls [23,24,25] and correlated angiogenesis in pterygium with tryptase-positive mast cell count [22]. Our study further expands on the potential role mast cells may play in the pathogenesis of pterygium by demonstrating GrB expression by these mast cells that are present in pterygium. However, as our group has recently addressed in a review paper [33], there are many cells that can express GrB, including immune cells such as T cells from various subsets, NK cells, basophils, and dendritic cells as well as non-immune cells such as keratinocytes and retinal pigment epithelial cells. Indeed, we noted some GrB-positive cells from pterygium specimens that did not co-express tryptase. Previous studies have demonstrated increased infiltration of CD4^+^ and CD8^+^ T lymphocytes in pterygium tissue when compared with normal conjunctival tissue, indicating that some of the GrB may come from other cell types as well [8,9,10]. Therefore, further studies are needed to better appreciate the breadth of sources for GrB expression in pterygium and their possibly synergistic role in disease pathogenesis.

Our study has some limitations to be addressed. First, the cross-sectional design of this study does not allow us to appreciate the temporal effects of GrB in pterygium. It would be a challenge to address this gap in human studies, but in vitro studies with varying degrees of UV exposure may help bridge this gap. Second, our study used a hypothesis-driven approach based on prior studies to assess the role of mast cells in GrB production in pterygium. However, some tryptase-negative cells expressing GrB were also found in our study, and it is unclear whether these cells are tryptase-negative mast cells or other cell types that are responsible for GrB expression. To this end, future work will focus on using fresh pterygium tissues surgically removed from patients to immunophenotype cells via multi-color flow cytometry. Third, our study was limited by the small sample size and could not comment on the potential differences between primary and recurrent pterygium. Likewise, while pterygium specimens were nasal excisions, our control specimens were either nasal or temporal excisions; thus, we could not comment on the potential differences between nasal and temporal tissue growth. Lastly, this current study focused on examining the cell type and location associated with GrB expression in pterygium in a semi-quantitative manner. Based on this finding, our future study will utilize a more quantitative approach such as enzyme-linked immunosorbent assay to provide a more nuanced understanding of the role of GrB in pterygium disease pathogenesis.

## 4. Materials and Methods

### 4.1. Tissue Collection

The study was approved by the University of British Columbia Clinical Research Ethics Board (H23-00712). Formalin-fixed, paraffin-embedded pterygium tissues (N = 8) were obtained from the Department of Pathology and Laboratory Medicine at Vancouver General Hospital (VGH) and University of British Columbia. Post-mortem conjunctival tissues from control eyes (age-matched controls) were obtained from the Eye Bank of British Columbia (N = 8). All 8 pterygium specimens were from male donors, while 6 age-matched control specimens were from male donors. The average donor age of the pterygium group was 59 (range 43–81), while that of the control group was 58 (range 38–74).

### 4.2. Immunofluorescence

Formalin-fixed, paraffin-embedded sections of human conjunctival tissues were sectioned at 6 μm thickness using a Leica RM2255 rotary microtome. Sectioned tissues were dried overnight at room temperature. Before immunostaining, sections were deparaffinized and rehydrated in xylene in decreasing concentrations from 100% ethanol to 50% ethanol. Antigen retrieval was achieved by incubation with Proteinase K for 30 min at room temperature followed by repeated washing with phosphate buffer saline (PBS). Endogenous peroxidase activity was quenched by incubating sections in 3% H_2_O_2_ solution for 10 min. Sections were washed in PBS and carefully vacuum-dried. Sections were then blocked with 3% normal goat serum for 20 min at room temperature and incubated overnight at 4 °C with a 1:100 dilution of rabbit anti-human GrB antibody (Rabbit polyclonal, ab4059 Abcam, UK). The following day, sections were incubated with a 1:500 dilution of goat anti-rabbit antibody (Fisher Scientific, Hampton, NH, USA) for 45 min at room temperature followed by washing with PBS. Sections were subsequently incubated with 1:500 dilution of DAPI for 10 min at room temperature and thoroughly washed with PBS on a shaker over an hour at room temperature. Negative control samples underwent the same treatment without primary antibody incubation.

For double immunofluorescence staining for GrB and tryptase, the samples underwent antigen retrieval by heating with citrate buffer for 10 min, followed by blocking with 3% normal goat serum (Vector Laboratories, Newark, CA, USA, S-1000-20) in 0.3% Triton X-100. The sections were then probed using primary antibody against tryptase (Mouse monoclonal, ab2378 Abcam, 1:100 dilution) overnight at 4 °C, followed by three PBS washes and incubation at room temperature for 45 min with a fluorescently tagged goat anti-mouse secondary antibody (Alexa fluor 546, Invitrogen, Waltham, MA, USA, 1:400 dilution). After thorough washing with PBS, samples were then incubated for 2 h at room temperature with primary antibody against GrB (Rabbit polyclonal, ab4059 Abcam, 1:100 dilution), followed by further PBS washes. Slides were then incubated for 45 min at room temperature with a fluorescently tagged goat anti-rabbit secondary antibody (Alexa fluor 488, Invitrogen, 1:500 dilution). In all cases, negative control sections were processed in parallel in an identical manner, except the primary antibody was omitted from the diluents. After additional washes, the nuclei were labeled with DAPI, and cross-sections were cover-slipped and imaged.

The Zeiss LSM 800 confocal microscope with Zen 2.6 Blue version software (Carl Zeiss, Germany) was used to visualize and capture fluorescent images under 20× and 40× magnification. Pterygium tissues were imaged using tiling at 40×, whereby the immunostained tissues were scanned in its entirety. This method allowed us to appreciate the distribution of signals in the tissue and to quantify the density of signals per given surface area. All settings on the confocal microscope were kept constant throughout the imaging sessions to compare the intensity of fluorescence signals between groups.

### 4.3. Quantification

Quantification of signals was performed using ImageJ (ImageJ bundled with Java 8, NIH, Madison, WI, USA). GrB-positive signals were manually counted by a single investigator, and functions within ImageJ were used to generate the total tissue surface area. Two immunostained sections were used for each donor to calculate an average density of signals per surface area.

### 4.4. Statistical Analyses

Statistical analyses were performed using Prism 9 (Prism v9.4.1, GraphPad Software, Boston, MA, USA). To compare two groups, Mann–Whitney was used for immunofluorescent analysis of human tissue samples. Statistical significance level was set at *p* < 0.05.

## 5. Conclusions

Our study shows that GrB expression is upregulated in pterygium tissues when compared to healthy, age-matched control conjunctival tissues and that many of these cells co-express tryptase. Further studies are warranted to investigate GrB as a possible therapeutic target in pterygium.

## Data Availability

The original contributions presented in the study are included in the article; further inquiries can be directed to the corresponding author.
